# Surface-based correlates of cognition along the Alzheimer's continuum in a memory clinic population

**DOI:** 10.3389/fneur.2023.1214083

**Published:** 2023-09-05

**Authors:** Michelle M. Coleman, Cierra M. Keith, Kirk Wilhelmsen, Rashi I. Mehta, Camila Vieira Ligo Teixeira, Mark Miller, Melanie Ward, Ramiro Osvaldo Navia, William T. McCuddy, Pierre-François D'Haese, Marc W. Haut

**Affiliations:** ^1^Rockefeller Neuroscience Institute, West Virginia University, Morgantown, WV, United States; ^2^Department of Behavioral Medicine and Psychiatry, West Virginia University, Morgantown, WV, United States; ^3^Department of Neurology, West Virginia University, Morgantown, WV, United States; ^4^Department of Neuroradiology, West Virginia University, Morgantown, WV, United States; ^5^Department of Medicine, West Virginia University, Morgantown, WV, United States; ^6^Department of Neuropsychology, St. Joseph Hospital and Medical Center, Barrow Neurological Institute, Phoenix, AZ, United States

**Keywords:** Alzheimer's disease, mild cognitive impairment, learning, memory, language, executive function, MRI

## Abstract

Composite cognitive measures in large-scale studies with biomarker data for amyloid and tau have been widely used to characterize Alzheimer's disease (AD). However, little is known about how the findings from these studies translate to memory clinic populations without biomarker data, using single measures of cognition. Additionally, most studies have utilized voxel-based morphometry or limited surface-based morphometry such as cortical thickness, to measure the neurodegeneration associated with cognitive deficits. In this study, we aimed to replicate and extend the biomarker, composite study relationships using expanded surface-based morphometry and single measures of cognition in a memory clinic population. We examined 271 clinically diagnosed symptomatic individuals with mild cognitive impairment (*N* = 93) and Alzheimer's disease dementia (*N* = 178), as well as healthy controls (*N* = 29). Surface-based morphometry measures included cortical thickness, sulcal depth, and gyrification index within the “signature areas” of Alzheimer's disease. The cognitive variables pertained to hallmark features of Alzheimer's disease including verbal learning, verbal memory retention, and language, as well as executive function. The results demonstrated that verbal learning, language, and executive function correlated with the cortical thickness of the temporal, frontal, and parietal areas. Verbal memory retention was correlated to the thickness of temporal regions and gyrification of the inferior temporal gyrus. Language was related to the temporal regions and the supramarginal gyrus' sulcal depth and gyrification index. Executive function was correlated with the medial temporal gyrus and supramarginal gyrus sulcal depth, and the gyrification index of temporal regions and supramarginal gyrus, but not with the frontal areas. Predictions of each of these cognitive measures were dependent on a combination of structures and each of the morphometry measurements, and often included medial temporal gyrus thickness and sulcal depth. Overall, the results demonstrated that the relationships between cortical thinning and cognition are widespread and can be observed using single measures of cognition in a clinically diagnosed AD population. The utility of sulcal depth and gyrification index measures may be more focal to certain brain areas and cognitive measures. The relative importance of temporal, frontal, and parietal regions in verbal learning, language, and executive function, but not verbal memory retention, was replicated in this clinic cohort.

## 1. Introduction

Alzheimer's disease (AD) is the most common cause of dementia in the elderly (1–5). Impairments in learning and the formation of new memories (2, 6), as well as progressive cognitive deficits in language (2, 3), are the hallmarks of this debilitating disease (7). Deficits in executive function are also commonly observed, particularly later in the disease course (2, 3). On the AD continuum, mild cognitive impairment (MCI) is where individuals are considered to be in a pre-dementia stage and often eventually progress to AD dementia (1–3). MRI with voxel-based morphometry has been used to examine the volumes of known regions of neurodegeneration in AD relative to cognitive deficits (2, 6). Atrophy occurs progressively from the hippocampus to the parietal and frontal lobes in AD (1, 2, 6, 8). Decreased volume, as a marker for cell death, in the mesial temporal lobe correlates strongly with learning and memory retention in AD (6). However, learning and memory retention have also been associated with volume loss in the other non-mesial temporal lobe, frontal and parietal regions (2). In addition to gray matter atrophy, white matter hyperintensities (WMH) often occur with AD and are associated with memory retrieval and other executive dysfunction (6). While voxel-based morphometry has been widely used to detect diffuse gray matter volume differences in AD, surface-based morphometry can measure cortical thickness and folding patterns to potentially demonstrate more subtle atrophic changes (1).

Surface-based cortical thinning as a proxy for atrophy in AD may be a more specific marker than volume-based measures (1–5, 7–9). In a seminal work, Dickerson et al. (8) identified cortical thinning in the following regions in both MCI due to AD and AD dementia: inferior temporal gyrus, middle temporal gyrus, temporal pole, middle frontal gyrus, superior frontal gyrus, precuneus, supramarginal gyrus, superior parietal lobule, and angular gyrus. Other authors have also linked these brain regions, referred to as the “signature areas” to the core cognitive deficits in AD (1, 7). While many studies have investigated cortical thickness as a marker of atrophy in Alzheimer's disease (1–5, 7, 8, 10), fewer studies have analyzed additional measures of surface morphometry such as sulcal depth and gyrification index [GI; (1, 3–5, 10, 11)]. Sulcal depth is defined as the distance of the cerebral hull to the outer surface of the cortex (12), whereas GI is the outer-to-inner cortex surface size ratio (1). Sulci widen and become more shallow with AD (5), and decreases in GI have been reported to be associated with stages of disease and progression of symptoms in AD (5, 10). GI and sulcal depth are possibly more sensitive than voxel-based morphometry for neurodegeneration or may help to further elucidate the cortical changes associated with cognitive decline in AD (1, 5).

Many studies examining surface morphometry have used the Alzheimer's Disease Neuroimaging Initiative [ADNI; (1, 2, 8, 10, 11, 13)], or similar large-scale biomarker-intensive studies (5, 7, 14). These large relatively homogenous research populations diagnosed with biomarker measures of both amyloid and tau often do not translate to the reality of heterogenous clinic populations. Whether surface morphometry measures are sensitive enough to detect meaningful associations between cortical atrophy and cognitive performance within a heterogenous clinical sample is less well understood. Large-scale studies also tend to use composite measures of cognition. However, in the clinical setting, providers have constraints on time and resources and may be less able to obtain the multiple measures needed to develop a composite score of cognition. Thus, establishing whether single measures of cognition can be used to find the same relationships would be beneficial.

This study aimed to investigate the relationship between cognition and surface-based morphometry measures with clinically diagnosed symptomatic patients with MCI or AD dementia among a community cohort. We used single measures to represent verbal learning, verbal memory retention, language, and executive function in hopes of encompassing the broad measure of each, only with a single cognitive measure. While these do not completely encompass each broad measure of cognition, they are used as representations of global measures. Alongside cortical thickness, we included additional measures of surface morphometry, sulcal depth, and GI. We hypothesized that greater thickness, depth, and gyrification would be associated with better verbal learning, verbal memory retention, language, and executive function in the signature areas of Alzheimer's disease (8).

## 2. Methods

### 2.1. Participants

**Figure 1:**
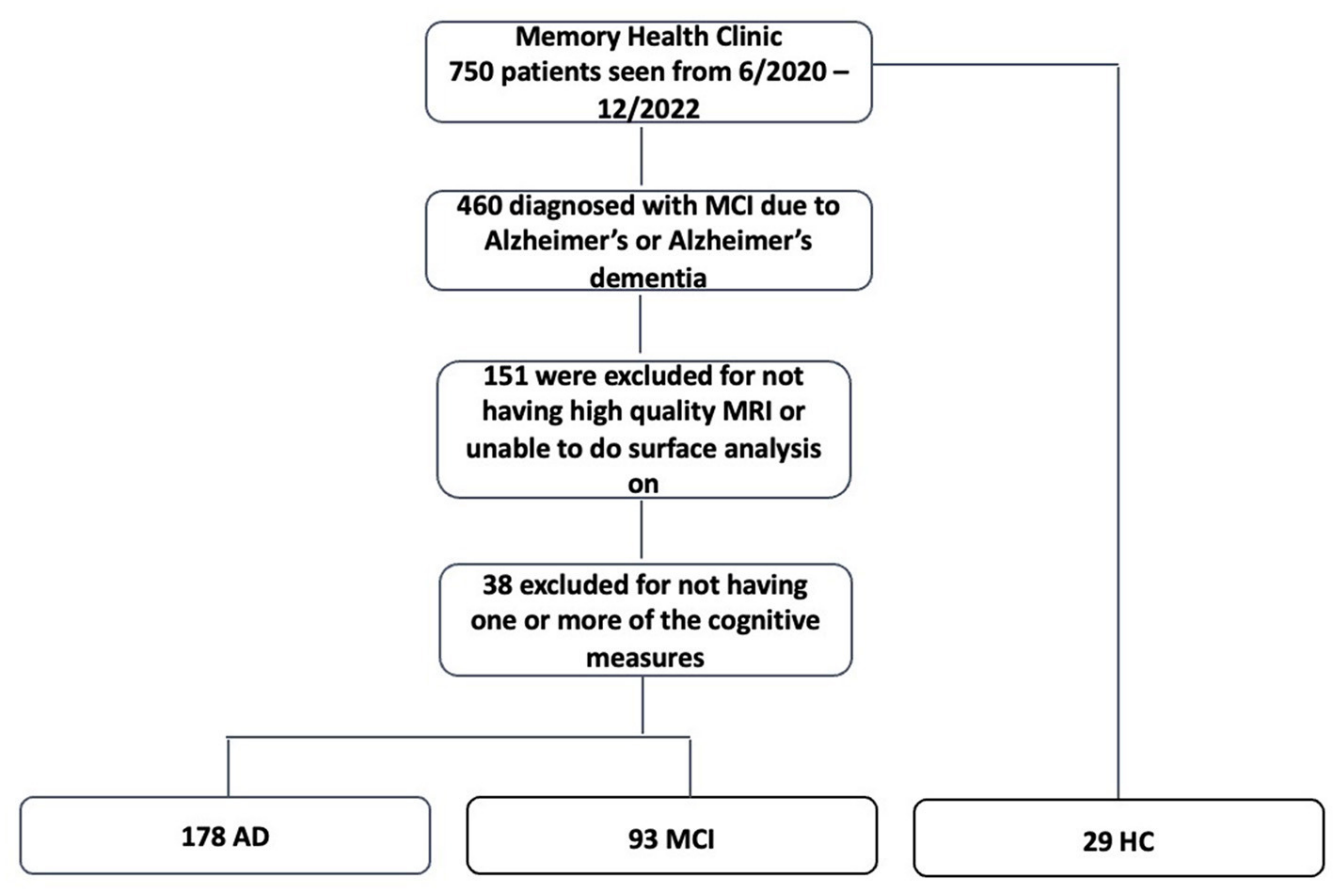
Flow chart demonstrating participant selection. MCI, mild cognitive impairment; MRI, magnetic resonance imaging; AD, Alzheimer's disease; HC, healthy controls.

Participants were 271 consecutive patients seen between June 2020 and December 2022 at an academic medical center memory clinic who met the diagnostic criteria for MCI or probable AD dementia using the National Institute on Aging-Alzheimer's Association criteria [NIA-AA; (14, 15)]. Twenty-nine additional patients who were concerned about their cognition but were not diagnosed with cognitive impairment were included as healthy controls (HC) (Figure 1). The diagnosis was achieved via the consensus of a multidisciplinary team, consisting of neurology, neuropsychology, neuroradiology, psychiatry, and geriatrics experts, that reviewed the patient's history, symptoms, neuroimaging, and neuropsychological evaluations. Completion of a neuropsychological evaluation with each cognitive measure and a high-quality, high-resolution structural MRI was required for inclusion in this study. The Institutional Review Board at West Virginia University approved the study, and all participants signed informed consent.

### 2.2. Measures

Neuropsychological evaluation of the patients included:

California Verbal Learning Test-II Short Form [CVLT-II SF; (16, 17)]: nine words from three categories are repeatedly presented with recall after each presentation and then after a 10-min delay. We used the total number of words recalled over the four learning trials as our single measure of verbal learning and then the percentage of words retained over the 10-min delay as our measure of verbal memory retention (6). A higher total number of words recalled indicates better performance. A higher score for percent retained translates to more storage of information.

Animal Naming (18) assessed semantic fluency, as a measure of language, by recording the number of animals named in 60 s. The total number of words was used as our single measure of language, where a higher score indicates better language performance.

Trails Making Tests part B [TMT-B; (19)]: is a widely used global measure of executive function where subjects must connect dots alternating between numbers and letters in order. This does not measure all aspects of executive function but was used here as a single measure of the executive aspect of cognition. The total time to completion of the task was used as a primary cognitive variable for executive function, where a higher score indicates lower executive functioning.

In addition, we have included several other variables that are present in most but not all participants to characterize the sample.

Mini-Mental State Examination [MMSE; (19)]: measures global mental status with a maximum score of 30.

Wide-Range Achievement Test, 4th Edition-Word Reading Subtest [WRAT-4-WR; (20)]: is a word reading task that was used to estimate premorbid intelligence, which has been previously done and validated as a measure of premorbid functioning (21, 22).

Functional Activities Questionnaire [FAQ; (23, 24)]: assesses impairment by activities of daily living that the caregiver can complete. A total score out of 30 was used with a higher score indicating more functional impairment.

Geriatric Depression Scale–short form [GDS; (21, 25)]: the total score was used as a measure of self-reported symptoms associated with depression with higher scores indicating greater depression.

Neuropsychiatric Inventory Questionnaire [NPI-Q; (22, 26)]: measures a number of neuropsychiatric symptoms. We used the total number of symptoms with a maximum of 12.

### 2.3. MRI data acquisition and processing

High-resolution structural MRI data were collected on most participants using a Siemens Prisma 3T scanner. T1 MPRAGE images were completed at a resolution of at least 1 × 1 × 1 mm (TR = 2,300 ms; TE = 2.26 ms) using a 20-channel head coil. A 48-channel head coil with a GE Architect 3T scanner was used on 26 patients to collect a T1 SPGR BRAVO image with a comparable resolution of 1 × 1 × 1 mm (TR = 8.5 ms; TE = 3.3 ms). No reliable difference was seen between Siemens and GE regarding image quality (IQR; F < 1). A T2 FLAIR 3D sequence with 1 mm resolution (TR = 6,000 ms; TE = 399 ms) was collected from most participants. Thirty-one participants had a lower resolution 2D FLAIR image with a 5-mm slice thickness. There was no difference between those who received higher versus lower resolution images for WMH volumes (F < 1).

**Figure 2:**
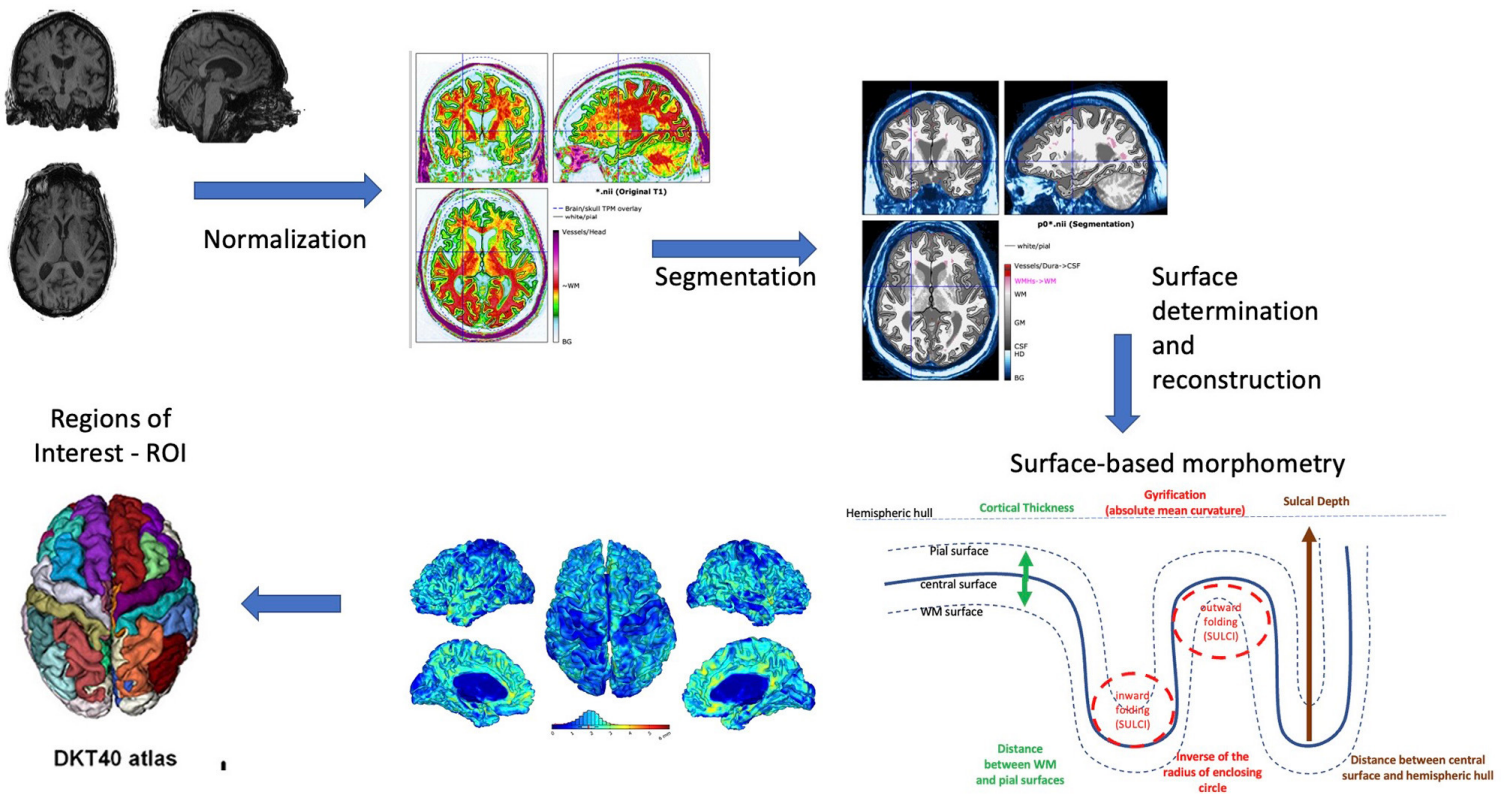
Surface-based morphometry explanation diagram.

Surface reconstruction was accomplished using the Computational Anatomical Toolbox (CAT12) (http://www.neuro.uni-jena.de/cat/) (27) run within the Statistical Parametric Mapping (SPM12) package (http://www.fil.ion.ucl.ac.uk/spm/software/spm12/) in the MATLAB environment (R2021b; https://www.mathworks.com). Patients were only included if their IQR was greater than or equal to 70 (Mean = 85.1 SD = 5.4). Standard preprocessing was completed without extra smoothing. Default parameters from CAT12 were used and included tissue segmentation, spatial registration, and surface creation and registration, as described previously (1, 28). Initial voxel-based preprocessing included denoising data, completed “unified segmentation” (29), skull stripping, determined left and right hemispheres, subcortex, and cerebellum, as well as local WMH (27). Projection-based thickness models were used for surface-based processing in CAT12 where the surface was spatially registered to the average Free Surfer template and included spatial smoothing (28, 30, 31). Projection-based thickness models used by CAT12 allow for topological defects to be accounted for and corrected (14). With CAT12, quicker but still reliable brain surface mapping is performed without reconstructing the entire surface such as with Free Surfer. Rather, CAT12 looks at the central surface using boundaries of gray matter to white matter and gray matter to cerebrospinal fluid [CSF; (14)]. The surface-based morphometry was applied to investigate features of the cortical surface. In brief, cortical thickness measures the width of the gray matter ribbon as the distance between its inner boundary (white matter surface) and outer boundary (pial surface) (28). Gyrification is calculated via the absolute mean curvature of the central surface (31), and sulcal depth is calculated as the distance from the central surface to the hemispheric hull (28) (Figure 2). The DKT40 atlas is an automated labeling system created by subdividing the human cerebral cortex into gyral-based regions of interested (32). In brief, using 40 MRI scans, 34 cortical regions of interested were identified manually in each of the individual hemispheres, and this information was encoded in the form of an atlas, that later was utilized to automatically label the regions (32). After segmentation, surface-based morphometry can determine various aspects of cortical shape, including cortical thickness, degree of gyral convolution (the gyrification index), and sulcal depth, after applying the DKT40 atlas.

The surface patterns of gray matter cortical thickness, sulcal depth, and gyrification index were investigated using the signature area region-of-interests typically involved in AD (8). The signature areas were defined using the DKT40 atlas (33). The DKT40 atlas cortical regions we included were the inferior temporal gyrus, middle temporal gyrus, temporal pole, rostral and caudal middle frontal gyrus (rostral and caudal were added to make the middle frontal gyrus), superior frontal gyrus, precuneus, supramarginal gyrus (separated from angular gyrus within inferior parietal lobule), superior parietal lobule, and inferior parietal lobule. The left and right hemispheres were averaged for each region.

White matter hyperintensity (WMH) volume was calculated using the lesion prediction algorithm from the Lesion Segmentation Toolbox (34) operating within SPM12. We used the total volume of WMH expressed in milliliters.

### 2.4. Statistical analysis

We conducted descriptive analyses of demographic variables, including age, education, sex, race, and estimated IQ, as well as clinical and MRI measures with frequencies, means, and standard deviations. Pearson's correlation was used to examine the relationship among our verbal learning, verbal memory retention, language, and executive function measures with each brain region for each surface measure. Cortical thickness is hypothesized to be positively associated with verbal learning, verbal memory retention, and language, where lower scores mean worse performance. Our prediction of the single measure of executive function is hypothesized to show a negative correlation where increased time (higher score) would indicate worse performance. Sulcal depth, where less depth means more flattening and thus atrophy, would show the same pattern as cortical thickness. Finally, GI, where augmented gyrification represents the increased degree of folding, should also demonstrate the same pattern of sulcal depth and cortical thickness. If a significant correlation between WMH and any of the primary cognitive measures was observed, we planned to repeat the correlations with WMH as a covariate. To correct for multiple comparisons, with three measures of the cortical surface and nine signature regions, we had 108 correlations. Therefore, we used a Bonferroni correction with a significance level of.05/108 = *p* < 0.0005. Given our sample size, a significant correlation will need to be = >0.2, with power equaling.44 under these parameters. Following Pearson correlations, multiple linear regressions were run, including all significant correlations as predictors for each of the cognitive variables.

## 3. Results

Sample characteristics are described in Table 1, including demographic and clinical information. Data presented in Table 1 show means for normally distributed data, and ranges and medians for data that are not normally distributed. The study sample included 300 participants (AD = 178, MCI = 93, HC = 29) with a majority being female (59%), well-educated (14.4 ± 2.7), and primarily white (97%). On average, participants did not endorse many symptoms of depression (GDS = 3.6 ± 2.9) but showed some other neuropsychiatric symptoms (NPI-Q = 3.9 ± 2.8). The sample showed generally mild impairment on cognitive screening (MMSE = 25.0 ± 3.6) and mild functional deficits (FAQ = 10.4 ± 8.2). While the averages shown in Table 1 are across the entire sample including healthy controls who did not show cognitive impairments, the clinical sample alone was nonetheless more mildly impaired. Table 2 presents the descriptive statistics for the cortical thickness, sulcal depth, and gyrification index, respectively, for the signature regions of interest.

**Table 1:** Characteristic demographic and cognitive data of the present sample.

**Measure**	**Mean (SD)**	**Range**	**Median**
Sex	F = 177/M = 123	–	–
Race	C = 291/B = 7/A = 2	–	–
Age	73.2 (7.8)	–	–
Handedness	R = 275/L = 25	–	–
Education	14.4 (2.7)	–	–
WMH	–	0.12–64.34	4.9
IQ estimate	–	55–145	99.0
MMSE	–	14–30	25.0
FAQ	–	0–28	9.0
GDS	–	0–13	3.0
NPI total	–	0–11	4.0
CVLT total	19.4 (6.4)	–	–
CVLT percent retained new	–	0–200	33.3
Animal naming	13.4 (5.4)	–	–
TMT-B	–	30–341	168.5

Characteristic data are demonstrated with means and standard deviations (SD) for normally distributed data, and ranges and medians for data that are not normally distributed (n = 300).

M, Male; F, Female; C, Caucasian; B, Black; A, Asian; R, Right; L, Left; WMH, White Matter Hyperintensity.

IQ Estimate from Wide-Range Achievement Test, 4th Edition-Word Reading Subtest (WRAT-4-WR; N = 296), Mini-Mental State Examination (MMSE; N = 297), Functional Activities Questionnaire (FAQ, N = 274), Geriatric Depression Scale (GDS; N = 292), Neuropsychiatric Inventory (NPI total symptoms; N = 248), California Verbal Learning Test-II Short Form (CVLT-II SF total words retained and percent retained of new words), Trails Making Test-B (TMT-B).

**Table 2:** Descriptive statistics on AD signatures areas' cortical thickness, sulcal depth, and gyrification index.

**Area**	**Cortical thickness mean (SD)**	**Sulcal depth mean (SD)**	**Gyrification index mean (SD)**
Inferior temporal gyrus	2.4 (0.2)	5.8 (0.5)	29.1 (1.0)
Medial temporal gyrus	2.5 (0.2)	5.3 (0.5)	28.8 (1.1)
Temporal pole	3.1 (0.5)	7.0 (0.9)	25.8 (1.7)
Middle frontal gyrus	2.2 (0.2)	7.5 (0.6)	29.3 (1.0)
Superior frontal gyrus	2.4 (0.2)	5.4 (0.5)	28.2 (0.8)
Precuneus	2.2 (0.2)	8.1 (0.8)	29.6 (0.9)
Supramarginal gyrus	2.3 (0.2)	10.0 (0.9)	28.5 (1.0)
Superior parietal lobule	2.0 (0.2)	8.2 (0.8)	28.6 (1.0)
Inferior parietal lobule	2.2 (0.2)	7.2 (0.7)	29.5 (1.0)

Characteristic AD signature regions from our sample are described here with means and standard deviations (SD) for the surface morphometry measures.

Most of the significant correlations observed in the analysis were consistent with our expectations. As shown in Table 3, performances on verbal learning and executive function tasks were positively and negatively correlated with the cortical thickness of all of the signature regions, respectively. As expected, verbal learning and language were positively related to cortical thickness, indicating better verbal learning, and language abilities were associated with a thicker cortex. Verbal memory retention was positively correlated with the thickness of each of the temporal regions, as well as the inferior parietal lobule. The language function measure was associated with the thickness in each of the signature areas as well. Notably, executive function showed a negative correlation, where higher cortical thickness was associated with a shorter completion time for the task. This result was expected as completion time was utilized as the cognitive measure and higher scores indicate poorer performance.

**Table 3:** Correlations between each of the surface morphometry measures of the nine signature areas with verbal learning, verbal memory retention, language, and executive function.

**Cortical thickness**				
**Area**	**Verbal learning**	**Verbal memory retention**	**Language**	**Executive function**
Inferior temporal gyrus	0.36^*^	0.22^*^	0.34^*^	−0.33^*^
	*p < * 0.001	*p < * 0.001	*p < * 0.001	*p < * 0.001
Medial temporal gyrus	0.41^*^	0.25^*^	0.40^*^	−0.38^*^
	*p < * 0.001	*p < * 0.001	*p < * 0.001	*p < * 0.001
Temporal pole	0.35^*^	0.29^*^	0.32^*^	−0.29^*^
	*p < * 0.001	*p < * 0.001	*p < * 0.001	*p < * 0.001
Middle frontal gyrus	0.28^*^	0.15	0.27^*^	−0.27^*^
	*p < * 0.001	*p =* 0.007	*p < * 0.001	*p < * 0.001
Superior frontal lobe	0.26^*^	0.15	0.24^*^	−0.27^*^
	*p < * 0.001	*p =* 0.011	*p < * 0.001	*p < * 0.001
Precuneus	0.32^*^	0.20	0.28^*^	−0.33^*^
	*p < * 0.001	*p =* 0.001	*p < * 0.001	*p < * 0.001
Supramarginal gyrus	0.33^*^	0.17	0.27^*^	−0.31^*^
	*p < * 0.001	*p =* 0.003	*p < * 0.001	*p < * 0.001
Superior parietal lobule	0.29^*^	0.16	0.27^*^	−0.29^*^
	*p < * 0.001	*p =* 0.006	*p < * 0.001	*p < * 0.001
Inferior parietal lobule	0.33^*^	0.21^*^	0.31^*^	−0.31^*^
	*p < * 0.001	*p < * 0.001	*p < * 0.001	*p < * 0.001
**Sulcal depth**				
**Area**	**Verbal learning**	**Verbal memory retention**	**Language**	**Executive function**
Inferior temporal gyrus	0.15	0.07	0.22^*^	−0.19
	*p =* 0.012	*p =* 0.243	*p < * 0.001	*p =* 0.001
Medial temporal gyrus	0.21^*^	0.03	0.25^*^	−0.29^*^
	*p < * 0.001	*p =* 0.559	*p < * 0.001	*p < * 0.001
Temporal pole	0.07	0.05	0.10	−0.09
	*p =* 0.258	*p =* 0.353	*p =* 0.071	*p =* 0.121
Middle frontal gyrus	0.02	0.02	0.12	−0.10
	*p =* 0.718	*p =* 0.706	*p =* 0.045	*p =* 0.089
Superior frontal lobe	−0.08	−0.07	0.00	−0.02
	*p =* 0.158	*p =* 0.215	*p =* 0.982	*p =* 0.794
Precuneus	0.01	0.04	0.09	−0.08
	*p =* 0.916	*p =* 0.539	*p =* 0.135	*p =* 0.172
Supramarginal gyrus	0.14	0.06	0.22^*^	−0.25^*^
	*p =* 0.018	*p =* 0.320	*p < * 0.001	*p < * 0.001
Superior parietal lobule	−0.03	−0.02	0.05	−0.07
	*p =* 0.671	*p =* 0.797	*p =* 0.371	*p =* 0.262
Inferior parietal lobule	0.12	0.04	0.16	−0.17
	*p =* 0.045	*p =* 0.456	*p =* 0.007	*p =* 0.003
Inferior temporal gyrus	−0.26^*^	−0.25^*^	−0.27^*^	0.26^*^
	*p < * 0.001	*p < * 0.001	*p < * 0.001	*p < * 0.001
Medial temporal gyrus	−0.16	−0.04	−0.20	0.23^*^
	*p =* 0.006	*p =* 0.487	*p =* 0.001	*p < * 0.001
Temporal pole	−0.26^*^	−0.16	−0.23^*^	0.24^*^
	*p < * 0.001	*p =* 0.007	*p < * 0.001	*p < * 0.001
Middle frontal gyrus	−0.01	−0.07	−0.11	0.09
	*p =* 0.804	*p =* 0.224	*p =* 0.052	*p =* 0.112
Superior frontal lobe	−0.01	−0.01	−0.08	0.07
	*p =* 0.853	*p =* 0.811	*p =* 0.147	*p =* 0.234
Precuneus	0.01	−0.03	0.12	−0.03
	*p =* 0.871	*p =* 0.652	*p =* 0.037	*p =* 0.589
Supramarginal gyrus	0.19	0.14	0.31^*^	−0.24^*^
	*p =* 0.001	*p =* 0.018	*p < * 0.001	*p < * 0.001
Superior parietal lobule	0.01	−0.03	0.02	0.02
	*p =* 0.921	*p =* 0.627	*p =* 0.793	*p =* 0.673
Inferior parietal lobule	0.11	0.03	0.14	−0.10
	*p =* 0.066	*p =* 0.559	*p =* 0.014	*p =* 0.072

Pearson's correlations ran at a multiple comparison's *p* < 0.005 corrected value for surface morphometry measures, cortical thickness, sulcal depth, and gyrification index, assessing signature areas for verbal learning, verbal memory retention, language, and executive function. ^*^p < 0.0005.

The complete analysis of the sulcal depth and GI correlations with brain regions is in Table 3. Performance on verbal learning was positively correlated with the medial temporal gyrus sulcal depth and negatively correlated with temporal pole GI as well as inferior temporal gyrus GI. Unexpectedly, verbal memory retention did not show any significant findings with sulcal depth but was correlated to inferior temporal GI. Language performance had significant positive correlations with inferior temporal and medial temporal gyrus sulcal depth, as well as supramarginal gyrus sulcal depth and GI. Language performance was negatively correlated with temporal pole and inferior temporal GI. Executive function was significantly positively correlated with temporal regions' GI, whereas medial temporal gyrus sulcal depth and supramarginal gyrus sulcal depth and GI were negatively correlated.

Because we observed a significant relationship between the volume of WMH and verbal learning (*r* = −0.21, *p* < 0.0005), language (*r* = −0.24, *p* < 0.0005), and executive function (*r* = 0.30, *p* < 0.0005), we reanalyzed the relationships between these variables and the cortical surface measures that were significant by controlling for WMH. Most of the measures remained significant after correcting for multiple comparisons (Supplementary Table 1).

Linear regressions were completed after assessing the correlations to determine predictors of each cognitive variable. Only significant correlations were entered into the regression analysis. Relative predictors of verbal learning in order of contribution were medial temporal gyrus thickness and medial temporal gyrus sulcal depth, followed by temporal pole GI; their combination most contributed to verbal learning (21% of the variance). The combination of temporal pole thickness and inferior temporal gyrus GI best predicted verbal memory retention (10% of the variance). For language, the combination of medial temporal gyrus thickness, supramarginal GI, and medial temporal gyrus sulcal depth was most predictive (27% of the variance). Executive function was best predicted by the combination of medial temporal gyrus thickness, supramarginal GI, and medial temporal gyrus sulcal depth (23% of the variance). Those findings are shown in Table 4.

**Table 4:** Stepwise regressions for verbal learning, verbal memory retention, language, and executive function.

**Predictors for verbal learning**						
Model	R	R^2^	SEE	Δ R ^2^	ΔF	Significance
Medial temporal gyrus thickness	0.412	0.170	5.8	0.170	60.9	< 0.001^*^
Medial temporal gyrus thickness, medial temporal gyrus sulcal depth	0.436	0.191	5.8	0.021	7.6	0.006
Medial temporal gyrus thickness, medial temporal gyrus sulcal depth, temporal pole GI	0.452	0.205	5.7	0.014	5.3	0.023
**Predictors for verbal memory retention**						
Model	R	R^2^	SEE	Δ R ^2^	ΔF	Significance
Temporal pole thickness	0.288	0.083	38.7	0.083	27.0	< 0.001^*^
Temporal pole thickness, inferior temporal gyrus GI	0.322	0.104	38.4	0.021	6.8	0.009
**Predictors for language**						
Model	R	R^2^	SEE	Δ R ^2^	ΔF	Significance
Medial temporal gyrus thickness	0.402	0.162	5.0	0.162	57.4	< 0.001^*^
Medial temporal gyrus thickness, supramarginal gyrus GI	0.505	0.255	4.7	0.093	37.1	< 0.001^*^
Medial temporal gyrus thickness, supramarginal gyrus GI, medial temporal gyrus sulcal depth	0.518	0.268	4.6	0.013	5.4	0.021
**Predictors for executive function**						
Model	R	R^2^	SEE	Δ R ^2^	ΔF	Significance
Medial temporal gyrus thickness	0.375	0.141	91.3	0.141	48.8	< 0.001^*^
Medial temporal gyrus thickness, supramarginal gyrus GI	0.442	0.195	88.5	0.054	20.0	< 0.001^*^
Medial temporal gyrus thickness, supramarginal gyrus GI, medial temporal gyrus sulcal depth	0.477	0.227	86.9	0.032	12.3	0.001

Linear regressions with significant correlations for each cognitive variable with significant surface morphometry measures.

GI, Gyrification index. ^*^p < 0.0005.

## 4. Discussion

The present study examined relationships between measures of cortical surface morphology and single measures of the typical cognitive deficits in a clinically diagnosed population of individuals with AD and MCI, alongside HC. Consistent with our hypotheses, we demonstrated that the decline of verbal learning, language, and executive function in MCI and AD is associated with cortical thinning in wide temporal, frontal, and parietal cortex areas. Contrary to our hypotheses, we did not observe the same diffuse relationships with verbal memory retention or as much with the other measures of surface morphometry, sulcal depth, and gyrification index.

The effects of atrophy on the Alzheimer's disease continuum appear to be widespread as most of these seemingly distinct functions are not localized to one brain area. Verbal learning, language, and executive function were related to the cortical thickness of signature temporal, frontal, and parietal regions. As cortical thickness has been previously described as a strong marker of the progression of AD (1, 2, 7, 8, 35), these findings were expected. Previous reports (1, 2, 7, 8) assessing cognitive performance across the continuum (i.e., healthy controls, early and late MCI, and AD dementia) have shown that the progression of symptoms and level of impairment correlates most with reductions in thickness. Verbal memory retention correlated with primarily temporal region thickness as well as inferior parietal lobule thickness. Typically, memory retention as a specific measure has almost exclusively been associated with the temporal lobes, including the hippocampus and entorhinal cortex (1, 2, 14). These areas of the temporal lobe were not included in our study; however, the temporal measures that did emerge give partial support to this discussion. In addition to memory, a study examining verbal fluency and cortical thickness (35), noted similar cortical thinning in the temporal and parietal regions strongly correlated with language function. These data were also collected in a memory disorders clinic; however, they did not parse out specific diagnoses and it was not just limited to individuals on the AD continuum. Widespread atrophy correlating with the executive function measure was consistent with other studies from the ADNI database (2, 36) and other biomarker databases (5, 7). A previous study demonstrating language and executive function associated with atrophy of frontal areas (2) used composite measures to demonstrate lower cognitive scores on language and executive functioning related to cortical thinning. Executive function measures used in their study tested a variety of other cognitive processes, including attention, that were not represented with the single measure utilized in the current study. As we were attempting to replicate findings without composite cognitive measures or biomarker data, this demonstrates the strength of our findings for the main cognitive deficits of the AD continuum, using single measures that broadly categorize these deficits. Specifically, our findings demonstrate the importance of the signature temporal, frontal, and parietal regions in verbal learning, language, and executive functioning in a clinical sample.

Our results demonstrated that white matter hyperintensities correlated with the frontal/executive measure, with an inverse relationship between WMH and cognition, which has been widely reported (37, 38). Importantly, the cortical thickness correlations with verbal learning, language, and executive function remained mostly significant even after controlling for WMH volume. This finding suggests that the changes in cortical thickness are primary, and while WMH volume is related to these cognitive functions in AD, it may exacerbate the deficits rather than produce them. Throughout the AD continuum, the mediation of WMH and executive function has been previously seen where those with lower hippocampal volumes have lower measures of executive function and more WMH burden (39), which in turn correlates with episodic memory performance. Our results replicated to demonstrate this cortical relationship as well but isolated to verbal learning, language, and executive functioning. While some correlations were lost when controlling for WMH, when the regressions were run, WMH was included in the analysis of verbal learning, language, and executive function due to its significance and was not demonstrated to predict in any of the variance for verbal learning and language. Executive function, however, as previously discussed, has the most relationship with WMH and as such was included in the variance but did not contribute as much as the previous inclusions.

Comparatively and unlike what was predicted, both sulcal depth and GI had limited findings. Sulcal depth of temporal regions and supramarginal gyrus only correlated with our cognitive variables. While no sulcal depth correlations were seen with verbal memory retention, the medial temporal gyrus sulcal depth specifically correlated with verbal learning, language, and executive function. The medial temporal gyrus sulcal depth was also predictive of each of those findings. The sulcal depth of the medial temporal lobe has been used previously (40) to differentiate people with amnestic MCI from controls; however, they discussed this being related to memory rather than aspects of cognition we found.

GI appeared to show more focal relationships, with correlations limited to the temporal regions and supramarginal gyrus. These correlations with certain measures may show its specificity, and GI has been previously described as more focal than the cortical thickness and sulcal depth (1). While Wu et al. (1) found a global reduction in sulcal depth progressively in the AD continuum, GI was only significantly different in three areas (right lateral orbitofrontal, left lingual, and left insula), showing a more focal nature for this measure. These other brain areas may play a role in the disease process, but they are not considered part of the signature areas for AD, which we examined. Thus, the core cognitive deficits may show more specific relationships with other brain regions not assessed in the current study. In contrast to those focal findings though, a previous study (5) showed that with an increase in symptom presentation from mild-to-more severe impairment on the MMSE, there is a more global decreased GI.

Interestingly, inferior temporal gyrus GI and verbal memory retention were negatively rather than positively correlated as predicted such that higher GI was associated with poorer verbal memory retention. This higher gyrification with the inferior temporal gyrus has been previously reported (10) and was remarked to occur potentially due to significant atrophy of the mesial temporal lobe. Hypergyrification was speculated to occur early in the disease continuum through neurogenesis as a compensatory result of this atrophy (10). When seen post-mortem in the hippocampus, the severity of the disease positively correlated with markers of neurogenesis (10), which supports our finding. For language and executive function, parietal regions' GI tended to have the opposite direction correlation than expected. With language, higher gyrification in the temporal and frontal areas correlated with lower language abilities, whereas it correlated with higher abilities in the parietal areas. With executive function, more gyrification in the temporal and frontal areas correlated with lower executive functioning (higher score), but higher executive functioning (lower score) in most parietal regions. As the parietal region is one of the early, noticeable atrophic changes seen on imaging, different gyrification patterns than anticipated by area may be reason for what has been seen here and in another study (10).

Interestingly, and not hypothesized, was the consistent necessity of each of these surface morphometry measures in predicting almost every cognitive variable. Verbal learning was best predicted by medial temporal gyrus thickness, sulcal depth, and GI of the temporal pole. A previous study (13) using another verbal learning measure found their top predictors for immediate learning were medial temporal structures. They (13) also found that their verbal memory retention measure was best predicted by the angular gyrus, hippocampus, and amygdala, not found or not studied here, respectively. This study, however, did not delve into the surface morphometry measures to determine what aspects may be involved. In the present study, verbal memory retention was predicted by temporal pole thickness and inferior temporal gyrus GI. Language was best predicted by medial temporal gyrus thickness, supramarginal GI, and medial temporal gyrus sulcal depth. Predictors for executive function included medial temporal gyrus thickness, supramarginal GI, and medial temporal sulcal depth. The relative contribution particularly of the thickness and sulcal depth of the medial temporal gyrus is highlighted in each of the cognitive variables, except verbal memory retention. The importance of the temporal lobe is still demonstrated with temporal pole thickness and inferior temporal gyrus GI predicting verbal memory retention. As the medial temporal area has been most studied in the AD population, this is to be expected. Additionally, the contribution of a combination of medial temporal gyrus thickness, sulcal depth, and supramarginal gyrus GI in predicting both language and executive function is highlighted here. The interplay of the various surface morphometry measures, cortical thickness, sulcal depth, and GI, each together contributing to the prediction of cognition is a unique finding of this study.

There are limitations to this study, including the lack of biomarker data, such as brain and CSF amyloid-beta and tau levels, as another diagnostic tool in terms of constraining the sample to the AD continuum. Biomarker analysis, while improving diagnostic specificity, is not yet the standard of care with clinical populations. Thus, our replication of the findings from ADNI and similar biomarker studies validates cortical thickness (1, 2, 10) without biomarker information, in clinical samples. Our population has similar demographics to the ADNI cohort in terms of education and lack of ethnic diversity but this lack of diversity limits generalization to the larger population on the AD continuum. Our limited findings related to sulcal depth and GI may be a function of a smaller sample size. However, if larger sample sizes are needed to detect these relationships, it is likely that the effects are smaller and more subtle, and that cortical thickness of the signature areas remains the most robust in characterizing cognitive deficits along the AD continuum. Future studies should look beyond the signature regions of AD, especially with a larger sample size. This analysis may show other regions, such as the insular cortex GI, which may be related to memory and language (1, 10).

## 5. Conclusion

This study used a clinically diagnosed sample of amnestic MCI and AD dementia participants, with healthy controls, to explore how brain surface morphometry measures correlate with cognitive functions measured by single clinical tests. Cortical thinning had the most robust relationships in the broad areas of temporal, frontal, and parietal lobes with individual measures of verbal learning, language, and executive function. The sulcal depth and GI measures were more specific and focal atrophic measures that correlated with cognition in a more limited manner. Predictors of cognition related to the AD continuum surrounded a combination of the AD signature areas and often included the medial temporal area. Predicting these aspects of cognition did not just comprise the cortical thickness of signature areas but often involved multiple surface morphometry measures. Overall, this study replicates and expands on previous findings, highlighting the robust cortical thickness correlations with cognitive function in clinically diagnosed patients with limited biomarker data. Our results provide further evidence of the importance of temporal, frontal, and parietal regions in verbal learning, language, and executive function in this population.

## Data availability statement

The data utilized in this article will be made available upon reasonable request to qualified individuals.

## Ethics statement

The studies involving humans were approved by West Virginia University Institutional Review Board. The studies were conducted in accordance with the local legislation and institutional requirements. The participants provided their written informed consent to participate in this study.

## Author contributions

MC: conceptualization, original draft, reviewing, and editing. CK: investigation, original draft, reviewing, and editing. KW: conceptualization, investigation, analysis, reviewing, and editing. RM, MM, MW, and RN: investigation, reviewing, and editing. CV: reviewing and editing. WM: data curation, reviewing, and editing. P-FD'H: data curation, investigation, analysis, reviewing, and editing. MH: conceptualization, investigation, analysis, original draft, reviewing, and editing. All authors contributed to the article and approved the submitted version.
