# Supplementary material for: Surface-based correlates of cognition along the Alzheimer's continuum in a memory clinic population
Source: Front Neurol. 2023 Sep 5;14:1214083. doi: 10.3389/fneur.2023.1214083 (PMC10508059; doi:10.3389/fneur.2023.1214083)
Supplement: Supplementary file 1 [file Table_1.docx]

**Supplemental 1.** Verbal learning, language, and executive function significant correlations with AD signature areas’ cortical thickness, sulcal depth and GI, covarying WMH.

| **Area** | **Verbal learning correlations** |
| --- | --- |
| Inferior temporal gyrus thickness | .31*  p<.001 |
| Inferior temporal gyrus GI | -.23*  p<.001 |
| Medial temporal gyrus thickness | .37*  p<.001 |
| Medial temporal gyrus sulcal depth | .19  p=.001 |
| Temporal pole thickness | .29*  p<.001 |
| Temporal pole GI | -.23*  p<.001 |
| Middle frontal gyrus thickness | .24*  p<.001 |
| Superior frontal gyrus thickness | .22*  p<.001 |
| Precuneus thickness | .29*  p<.001 |
| Supramarginal gyrus thickness | .27*  p<.001 |
| Superior parietal lobule thickness | .26*  p<.001 |
| Inferior parietal lobule thickness | .29*  p<.001 |
| **Area** | **Language correlations** |
| Inferior temporal gyrus thickness | .29*  p<.001 |
| Inferior temporal gyrus sulcal depth | .22*  p<.001 |
| Inferior temporal gyrus GI | -.24*  p<.001 |
| Medial temporal gyrus thickness | .35*  p<.001 |
| Medial temporal gyrus sulcal depth | .23*  p<.001 |
| Temporal pole thickness | .25*  p<.001 |
| Temporal pole GI | -.19  p=.001 |
| Middle frontal gyrus thickness | .21*  p<.001 |
| Superior frontal gyrus thickness | .18  p=.002 |
| Precuneus thickness | .24*  p<.001 |
| Supramarginal gyrus thickness | .20  p=.001 |
| Supramarginal sulcal depth | .20*  p<.001 |
| Supramarginal gyrus GI | .29*  p<.001 |
| Superior parietal lobule thickness | .23*  p<.001 |
| Inferior parietal lobule thickness | .26*  p<.001 |
| **Area** | **Executive function correlations** |
| Inferior temporal gyrus thickness | -.25*  p<.001 |
| Inferior temporal gyrus GI | .22*  p<.001 |
| Medial temporal gyrus thickness | -.31*  p<.001 |
| Medial temporal gyrus sulcal depth | -.27*  p<.001 |
| Medial temporal gyrus GI | .21*  p<.001 |
| Temporal pole thickness | -.19  p=.001 |
| Temporal pole GI | .20  p=.001 |
| Middle frontal gyrus thickness | -.20*  p<.001 |
| Superior frontal gyrus thickness | -.20  p=.001 |
| Precuneus thickness | -.27*  p<.001 |
| Supramarginal gyrus thickness | -.23*  p<.001 |
| Supramarginal gyrus sulcal depth | -.23*  p<.001 |
| Supramarginal gyrus GI | -.22*  p<.001 |
| Superior parietal lobule thickness | -.24*  p<.001 |
| Inferior parietal lobule thickness | -.25*  p<.001 |

*Covarying out White Matter Hyperintensities (WMH) from the significant findings of verbal learning, language, and executive function on surface morphometry relationships.*

*Gyrification index (GI).*

**p<.0005*
